# *N*-(2,6-Dichloro­phen­yl)-5-methyl-1,2-oxazole-4-carboxamide monohydrate

**DOI:** 10.1107/S1600536811044734

**Published:** 2011-11-05

**Authors:** De-Cai Wang, Liang-Cheng Huang, Hua-Quan Liu, Yu-Ran Peng, Jun-Song Song

**Affiliations:** a State Key Laboratory of Materials-Oriented Chemical Engineering, School of Pharmaceutical Sciences, Nanjing University of Technology, Xinmofan Road No. 5 Nanjing, Nanjing 210009, People’s Republic of China

## Abstract

In the title compound, C_11_H_8_Cl_2_N_2_O_2_·H_2_O, the dihedral angle between the benzene and isoxazole rings is 59.10 (7)°. In the crystal, the components are linked by N—H⋯O and O—H⋯O hydrogen bonds into a three-dimensional network. The crystal structure is further stabilized by π–π stacking inter­actions [centroid–centroid distance = 3.804 (2) Å].

## Related literature

The title compound was synthesised as a new and potent immunomodulating leflunomide {systematic name: 5-methyl-*N*-[4-(trifluoro­meth­yl)phen­yl]-isoxazole-4-carboxamide} ana­log (Huang *et al.*, 2003 ▶). For the application of leflunomide in the treatment of rheumatoid arthritis, see: Shaw *et al.* (2011 ▶); Schattenkirchner *et al.* (2000 ▶).
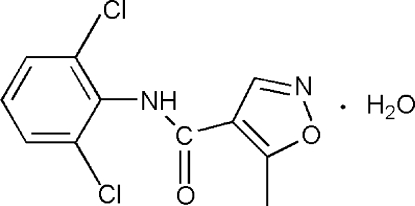


## Experimental

### 

#### Crystal data

C_11_H_8_Cl_2_N_2_O_2_·H_2_O
*M* *_r_* = 289.11
Orthorhombic, 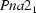

*a* = 12.047 (2) Å
*b* = 8.2290 (16) Å
*c* = 13.086 (3) Å
*V* = 1297.3 (4) Å^3^
*Z* = 4
Mo *K*α radiation
μ = 0.50 mm^−1^
*T* = 293 K
0.30 × 0.20 × 0.10 mm

#### Data collection

Enraf–Nonius CAD-4 diffractometer
Absorption correction: ψ scan (*SADABS*; Sheldrick, 1996 ▶) *T*_min_ = 0.864, *T*_max_ = 0.952
2333 measured reflections
2333 independent reflections
1906 reflections with *I* > 2σ(*I*)
3 standard reflections every 200 reflections  intensity decay: 1%

#### Refinement

*R*[*F* ^2^ > 2σ(*F* ^2^)] = 0.045
*wR*(*F* ^2^) = 0.118
*S* = 1.00
2333 reflections
164 parameters
1 restraint
H-atom parameters constrained
Δρ_max_ = 0.19 e Å^−3^
Δρ_min_ = −0.18 e Å^−3^
Absolute structure: Flack (1983 ▶), 1107 Friedel pairs
Flack parameter: 0.04 (9)

### 

Data collection: *CAD-4 EXPRESS* (Enraf–Nonius, 1994 ▶); cell refinement: *CAD-4 EXPRESS*; data reduction: *XCAD4* (Harms & Wocadlo,1995 ▶); program(s) used to solve structure: *SHELXS97* (Sheldrick, 2008 ▶); program(s) used to refine structure: *SHELXL97* (Sheldrick, 2008 ▶); molecular graphics: *SHELXTL* (Sheldrick, 2008 ▶); software used to prepare material for publication: *SHELXTL*.

## Supplementary Material

Crystal structure: contains datablock(s) I, global. DOI: 10.1107/S1600536811044734/gw2111sup1.cif

Structure factors: contains datablock(s) I. DOI: 10.1107/S1600536811044734/gw2111Isup2.hkl

Supplementary material file. DOI: 10.1107/S1600536811044734/gw2111Isup3.cml

Additional supplementary materials:  crystallographic information; 3D view; checkCIF report

## Figures and Tables

**Table 1:** Hydrogen-bond geometry (Å, °)

*D*—H⋯*A*	*D*—H	H⋯*A*	*D*⋯*A*	*D*—H⋯*A*
N1—H1*A*⋯O*W*^i^	0.86	2.07	2.897 (4)	161
O*W*—H*WB*⋯O1^ii^	0.85	2.00	2.839 (3)	168

Symmetry codes: (i) 
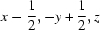
; (ii) 
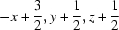
.

**Fig. 1.**
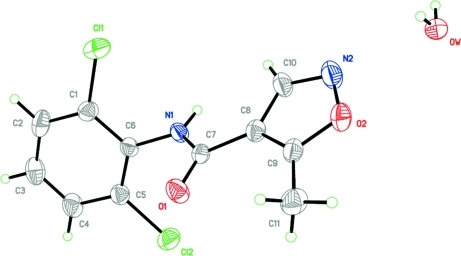
The structure of the title compound, showing the atomic numbering scheme. Non-H atoms are shown with 30% probability displacement ellipsoids.

## supplementary crystallographic information

### Comment

Leflunomide is one of the most effective isoxazole-containing heterocyclic disease modifying anti-rheumatic drugs for treating rheumatoid arthritis(Shaw *et al.*, 2011; Schattenkirchner *et al.*, 2000). The title compound 5-methyl-*N*-(2,6-dichlorophenyl)isoxazole -4-carboxamide monohydrate,(I), was synthesized as a novel and potent immunomodulating leflunomide analog (Huang, *et al.*, 2003). We report herein the crystal structure of the title compound.

As illustrated in Fig. 1, the molecular structure of the title compound is not planar and consists of one 5-methyl-*N*-(2,6-dichlorophenyl)isoxazole-4-carboxamide molecule and one solvate water molecule. The dihedral angle between the C1—C6 benzene and the C8—C10/N2/O2 isoxazole ring is 59.10 (7) °. The central nitrogen atom (N1) and carbon atom (C7) are nearly coplanar with the benzene ring and the benzoyl rings[N1—C6—C5—C4 torsion angles = -178.5 (3) ° and C7—C8—C9—O2 torsion angles = -179.2 (3) °], respectively. The length of the C10=N2 double bond is 1.299 (5) Å, slightly longer than standard 1.28 Å value of a C=N double bond. The crystal structure is stabilized by N—H···O and O—H···O hydrogen bonds (Table 1), which is further stabilized by /*p*-/p stacking interactions.

### Experimental

A solution of 0.005 mole of 5-methylisoxazole-4-carboxylic acid chloride (0.73 g) in 2 ml of acetonitrile is added dropwise, while stirring,to 0.01 mole of 2,6-dichloroaniline(1.62 g),dissolved in 15 ml of acetonitrile at room temperature.After stirring for 20 minutes,the precipitated 2,6-dichloroaniline hydrochloride is filtered off and washed with 10 ml portions of acetonitrile,and the combined filtrates are concentrated under reduced pressure.10.6 g(78.5% of theory) of white crytalline 5-methyl-*N*-(2,6-dichlorophenyl)isoxazole-4-carboxamide are thus obtained. Crystals of (I) suitable for X-ray diffraction were obtained by slow evaporation of an methylbenzene solution.

### Refinement

H atoms of the water molecule were located in a difference Fourier map and refined as riding with O—H = 0.85 Å, with *U*_iso_(H) = 1.5 *U*_eq_. Carbon and nitrogen bound H atoms were placed at calculated positions and were treated as riding on the parent C or N atoms with C—H = 0.96 (methyl), 0.97 (methylene) and N—H = 0.86 Å, *U*_iso_(H) = 1.2 or 1.5 *U*_eq_(C, N).

### Crystal data

**Table:**

C_11_H_8_Cl_2_N_2_O_2_·H_2_O	*D*_x_ = 1.480 Mg m^−^^3^
*M**_r_* = 289.11	Mo *K*α radiation, λ = 0.71073 Å
Orthorhombic, *P**n**a*2_1_	Cell parameters from 25 reflections
*a* = 12.047 (2) Å	θ = 9–13°
*b* = 8.2290 (16) Å	µ = 0.50 mm^−^^1^
*c* = 13.086 (3) Å	*T* = 293 K
*V* = 1297.3 (4) Å^3^	Block, white
*Z* = 4	0.30 × 0.20 × 0.10 mm
*F*(000) = 592	

### Data collection

**Table:**

Enraf–Nonius CAD-4 diffractometer	1906 reflections with *I* > 2σ(*I*)
Radiation source: fine-focus sealed tube	*R*_int_ = 0.000
graphite	θ_max_ = 25.4°, θ_min_ = 3.0°
ω/2θ scans	*h* = 0→14
Absorption correction: ψ scan (*SADABS*; Sheldrick, 1996)	*k* = −9→0
*T*_min_ = 0.864, *T*_max_ = 0.952	*l* = −15→15
2333 measured reflections	3 standard reflections every 200 reflections
2333 independent reflections	intensity decay: 1%

### Refinement

**Table:**

Refinement on *F*^2^	Hydrogen site location: inferred from neighbouring sites
Least-squares matrix: full	H-atom parameters constrained
*R*[*F*^2^ > 2σ(*F*^2^)] = 0.045	*w* = 1/[σ^2^(*F*_o_^2^) + (0.073*P*)^2^] where *P* = (*F*_o_^2^ + 2*F*_c_^2^)/3
*wR*(*F*^2^) = 0.118	(Δ/σ)_max_ < 0.001
*S* = 1.00	Δρ_max_ = 0.19 e Å^−^^3^
2333 reflections	Δρ_min_ = −0.18 e Å^−^^3^
164 parameters	Extinction correction: *SHELXL97* (Sheldrick, 2008), Fc^*^=kFc[1+0.001xFc^2^λ^3^/sin(2θ)]^-1/4^
1 restraint	Extinction coefficient: 0.038 (3)
Primary atom site location: structure-invariant direct methods	Absolute structure: Flack (1983), 1107 Friedel pairs
Secondary atom site location: difference Fourier map	Flack parameter: 0.04 (9)

### Special details

**Table:**

Geometry. All e.s.d.'s (except the e.s.d. in the dihedral angle between two l.s. planes) are estimated using the full covariance matrix. The cell e.s.d.'s are taken into account individually in the estimation of e.s.d.'s in distances, angles and torsion angles; correlations between e.s.d.'s in cell parameters are only used when they are defined by crystal symmetry. An approximate (isotropic) treatment of cell e.s.d.'s is used for estimating e.s.d.'s involving l.s. planes.
Refinement. Refinement of *F*^2^ against ALL reflections. The weighted *R*-factor *wR* and goodness of fit *S* are based on *F*^2^, conventional *R*-factors *R* are based on *F*, with *F* set to zero for negative *F*^2^. The threshold expression of *F*^2^ > σ(*F*^2^) is used only for calculating *R*-factors(gt) *etc*. and is not relevant to the choice of reflections for refinement. *R*-factors based on *F*^2^ are statistically about twice as large as those based on *F*, and *R*- factors based on ALL data will be even larger.

### Fractional atomic coordinates and isotropic or equivalent isotropic displacement parameters (Å^2^)

**Table:**

	*x*	*y*	*z*	*U*_iso_*/*U*_eq_	
Cl1	0.34861 (10)	−0.10973 (15)	0.99733 (8)	0.0766 (4)	
N1	0.4813 (2)	0.0638 (3)	0.8429 (2)	0.0426 (6)	
H1A	0.5009	0.1294	0.8907	0.051*	
O1	0.5393 (2)	−0.1120 (4)	0.7213 (2)	0.0572 (7)	
C1	0.2951 (3)	−0.0268 (5)	0.8868 (3)	0.0482 (9)	
Cl2	0.40753 (8)	0.20917 (15)	0.64182 (8)	0.0679 (3)	
N2	0.8178 (3)	0.0689 (5)	0.9349 (3)	0.0695 (10)	
C2	0.1817 (3)	−0.0353 (5)	0.8670 (4)	0.0654 (12)	
H2B	0.1340	−0.0829	0.9142	0.078*	
O2	0.85611 (19)	−0.0172 (4)	0.8485 (2)	0.0633 (8)	
C3	0.1411 (3)	0.0275 (6)	0.7769 (4)	0.0689 (12)	
H3A	0.0654	0.0213	0.7631	0.083*	
C4	0.2092 (3)	0.0973 (5)	0.7089 (4)	0.0605 (10)	
H4A	0.1807	0.1369	0.6478	0.073*	
C5	0.3222 (3)	0.1113 (4)	0.7288 (3)	0.0479 (8)	
C6	0.3673 (3)	0.0494 (4)	0.8180 (3)	0.0405 (7)	
C7	0.5598 (3)	−0.0226 (4)	0.7937 (2)	0.0400 (7)	
C8	0.6743 (3)	−0.0017 (4)	0.8333 (3)	0.0447 (8)	
C9	0.7686 (3)	−0.0582 (4)	0.7899 (3)	0.0474 (8)	
C10	0.7108 (3)	0.0774 (5)	0.9239 (3)	0.0579 (10)	
H10A	0.6636	0.1291	0.9698	0.069*	
C11	0.7943 (3)	−0.1487 (6)	0.6956 (3)	0.0623 (11)	
H11A	0.8728	−0.1679	0.6919	0.094*	
H11B	0.7712	−0.0863	0.6374	0.094*	
H11C	0.7558	−0.2508	0.6962	0.094*	
OW	0.9991 (2)	0.2436 (4)	1.02747 (19)	0.0577 (7)	
HWA	0.9856	0.1441	1.0398	0.069*	
HWB	0.9882	0.3005	1.0807	0.069*	

### Atomic displacement parameters (Å^2^)

**Table:**

	*U*^11^	*U*^22^	*U*^33^	*U*^12^	*U*^13^	*U*^23^
Cl1	0.0827 (8)	0.0957 (8)	0.0513 (6)	−0.0078 (6)	0.0037 (5)	0.0203 (6)
N1	0.0319 (13)	0.0569 (17)	0.0391 (14)	0.0007 (12)	−0.0025 (12)	−0.0103 (13)
O1	0.0431 (13)	0.0740 (18)	0.0546 (16)	0.0039 (13)	−0.0028 (12)	−0.0211 (14)
C1	0.045 (2)	0.055 (2)	0.0453 (19)	0.0018 (16)	0.0044 (16)	0.0008 (17)
Cl2	0.0573 (6)	0.0872 (7)	0.0594 (6)	−0.0051 (5)	0.0004 (5)	0.0229 (5)
N2	0.0433 (19)	0.087 (3)	0.078 (3)	−0.0051 (17)	−0.0078 (16)	−0.007 (2)
C2	0.041 (2)	0.069 (3)	0.086 (3)	−0.0103 (18)	0.016 (2)	−0.004 (2)
O2	0.0347 (14)	0.080 (2)	0.0756 (19)	0.0004 (12)	0.0016 (14)	0.0016 (16)
C3	0.035 (2)	0.077 (3)	0.094 (4)	−0.001 (2)	−0.008 (2)	−0.001 (3)
C4	0.039 (2)	0.070 (3)	0.073 (3)	0.0044 (19)	−0.0099 (19)	0.005 (2)
C5	0.0416 (18)	0.051 (2)	0.051 (2)	−0.0009 (16)	−0.0008 (15)	−0.0015 (17)
C6	0.0289 (15)	0.0504 (18)	0.0423 (17)	0.0050 (13)	−0.0009 (13)	−0.0030 (14)
C7	0.0285 (16)	0.0519 (19)	0.0397 (18)	0.0010 (14)	0.0024 (13)	0.0005 (16)
C8	0.0377 (17)	0.0455 (19)	0.051 (2)	0.0009 (14)	0.0019 (16)	0.0037 (17)
C9	0.0363 (19)	0.050 (2)	0.055 (2)	−0.0010 (16)	0.0024 (16)	0.0094 (17)
C10	0.038 (2)	0.077 (3)	0.059 (2)	−0.0035 (18)	−0.0050 (18)	−0.007 (2)
C11	0.053 (2)	0.071 (3)	0.063 (2)	0.0140 (19)	0.010 (2)	0.001 (2)
OW	0.0544 (14)	0.0756 (18)	0.0430 (12)	−0.0051 (13)	0.0021 (12)	−0.0017 (12)

### Geometric parameters (Å, °)

**Table:**

Cl1—C1	1.724 (4)	C3—H3A	0.9300
N1—C7	1.347 (4)	C4—C5	1.391 (5)
N1—C6	1.416 (4)	C4—H4A	0.9300
N1—H1A	0.8600	C5—C6	1.384 (5)
O1—C7	1.224 (4)	C7—C8	1.483 (4)
C1—C2	1.392 (5)	C8—C9	1.352 (5)
C1—C6	1.400 (5)	C8—C10	1.423 (5)
Cl2—C5	1.733 (4)	C9—C11	1.474 (5)
N2—C10	1.299 (5)	C10—H10A	0.9300
N2—O2	1.412 (5)	C11—H11A	0.9600
C2—C3	1.378 (6)	C11—H11B	0.9600
C2—H2B	0.9300	C11—H11C	0.9600
O2—C9	1.347 (4)	OW—HWA	0.8500
C3—C4	1.340 (6)	OW—HWB	0.8499
			
C7—N1—C6	121.8 (3)	C5—C6—N1	123.0 (3)
C7—N1—H1A	119.1	C1—C6—N1	119.4 (3)
C6—N1—H1A	119.1	O1—C7—N1	123.0 (3)
C2—C1—C6	120.8 (4)	O1—C7—C8	121.9 (3)
C2—C1—Cl1	120.2 (3)	N1—C7—C8	115.2 (3)
C6—C1—Cl1	119.0 (3)	C9—C8—C10	104.4 (3)
C10—N2—O2	105.2 (3)	C9—C8—C7	126.5 (3)
C3—C2—C1	119.3 (4)	C10—C8—C7	129.2 (3)
C3—C2—H2B	120.4	O2—C9—C8	109.4 (3)
C1—C2—H2B	120.4	O2—C9—C11	116.0 (3)
C9—O2—N2	109.0 (3)	C8—C9—C11	134.6 (4)
C4—C3—C2	120.7 (4)	N2—C10—C8	112.0 (4)
C4—C3—H3A	119.6	N2—C10—H10A	124.0
C2—C3—H3A	119.6	C8—C10—H10A	124.0
C3—C4—C5	120.7 (4)	C9—C11—H11A	109.5
C3—C4—H4A	119.6	C9—C11—H11B	109.5
C5—C4—H4A	119.6	H11A—C11—H11B	109.5
C6—C5—C4	120.8 (4)	C9—C11—H11C	109.5
C6—C5—Cl2	119.5 (3)	H11A—C11—H11C	109.5
C4—C5—Cl2	119.7 (3)	H11B—C11—H11C	109.5
C5—C6—C1	117.6 (3)	HWA—OW—HWB	110.3
			
C6—C1—C2—C3	2.1 (6)	C7—N1—C6—C1	109.6 (4)
Cl1—C1—C2—C3	−178.1 (4)	C6—N1—C7—O1	4.0 (5)
C10—N2—O2—C9	−0.8 (4)	C6—N1—C7—C8	−176.2 (3)
C1—C2—C3—C4	−0.5 (7)	O1—C7—C8—C9	8.6 (6)
C2—C3—C4—C5	−1.5 (7)	N1—C7—C8—C9	−171.2 (3)
C3—C4—C5—C6	1.8 (6)	O1—C7—C8—C10	−170.2 (4)
C3—C4—C5—Cl2	−177.5 (4)	N1—C7—C8—C10	9.9 (6)
C4—C5—C6—C1	−0.2 (5)	N2—O2—C9—C8	0.6 (4)
Cl2—C5—C6—C1	179.1 (3)	N2—O2—C9—C11	179.7 (3)
C4—C5—C6—N1	−178.5 (3)	C10—C8—C9—O2	−0.2 (4)
Cl2—C5—C6—N1	0.8 (5)	C7—C8—C9—O2	−179.2 (3)
C2—C1—C6—C5	−1.8 (5)	C10—C8—C9—C11	−179.0 (4)
Cl1—C1—C6—C5	178.5 (3)	C7—C8—C9—C11	1.9 (7)
C2—C1—C6—N1	176.6 (3)	O2—N2—C10—C8	0.7 (5)
Cl1—C1—C6—N1	−3.1 (5)	C9—C8—C10—N2	−0.3 (5)
C7—N1—C6—C5	−72.1 (4)	C7—C8—C10—N2	178.7 (4)

### Hydrogen-bond geometry (Å, °)

**Table:**

*D*—H···*A*	*D*—H	H···*A*	*D*···*A*	*D*—H···*A*
N1—H1A···OW^i^	0.86	2.07	2.897 (4)	161.
OW—HWB···O1^ii^	0.85	2.00	2.839 (3)	168.

Symmetry codes: (i) *x*−1/2, −*y*+1/2, *z*; (ii) −*x*+3/2, *y*+1/2, *z*+1/2.
